# Melbourne Epidemiological Study of Childhood Asthma (MESCA)

**DOI:** 10.1007/s10654-026-01377-3

**Published:** 2026-03-18

**Authors:** Dinh S Bui, Andrew Tai, Jiacheng Liu, Jennifer L Perret, Caroline J Lodge, Nur Sabrina Idrose, Mary Roberts, Colin Robertson, Shyamali C Dharmage

**Affiliations:** 1 Allergy and Lung Health Unit, Melbourne School of Population and Global Health, Melbourne University, Melbourne, VIC, Australia; 2 Respiratory and Sleep Department, Women’s and Children’s Hospital, Adelaide, SA Australia; 3 Adelaide Medical School, University of Adelaide, Adelaide, SA Australia; 4 Melbourne Royal Children Hospital, Melbourne, VIC Australia

**Keywords:** Cohort profile, Childhood asthma, COPD, Lung function, Allergy

## Abstract

The Melbourne Epidemiological Study of Childhood Asthma (MESCA) is one of the longest-running respiratory studies in the world. The study aimed to determine the prevalence and describe the natural history of childhood asthma and wheezy bronchitis. MESCA started in 1964, when four asthma/wheeze groups and a control group, all aged 7, were recruited and have been followed into their seventh decade. The study has collected unique, repeated data on asthma, respiratory symptoms, and lung function over seven decades. It has provided critical insights into the natural history and long-term outcomes of childhood asthma. MESCA is the first prospective study to provide robust evidence for the link between childhood asthma and the development of COPD. Participants are now entering their seventh decade of life, and their rich, lifetime data provides a unique opportunity to investigate a wide range of outcomes, including multimorbidity and healthy aging.

**Supplementary Information:**

The online version contains supplementary material available at 10.1007/s10654-026-01377-3.

## Introduction

### The study set-up

The Melbourne Epidemiological Study of Childhood Asthma (MESCA) also known as the Melbourne Asthma Study, was initiated in 1964 by the late Howard Williams of the Royal Children’s Hospital, Melbourne, with the initial aim of determining the prevalence and describing the natural history of childhood asthma and wheezy bronchitis. In the early 1960 s, wheezy bronchitis was referred to as episodes of wheezing associated with symptoms of an underlying respiratory tract infection. It was hypothesized at the time that wheezy bronchitis and asthma were part of the same disorder, differing in their spectra of severity and long-term outcomes. It was thought that using the term wheezy bronchitis resulted in inappropriate treatment of wheezing episodes in children with antibiotics rather than anti-asthma medications. Follow-up studies of this cohort were conducted 8 times over six decades to address major knowledge gaps about wheeze and asthma including (1) probability of remission/persistence of wheeze into adolescence and adulthood and their determinants/predictors, (2) how the pattern of asthma in childhood predicted the pattern in adult life, (3) the long-term relationship of asthma with lung function, particularly the risk of developing fixed airways disease and (4) the relationship between features of allergy and the life course of asthma.

## Methods and materials

### Who is in the cohort, how often have they been followed up, and the amount of attrition over time

**Fig. 1:**
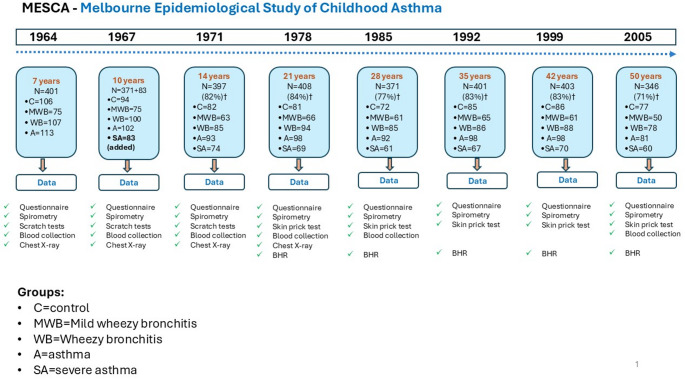
Flow chart of the study. †The percentages from ages 14 to 50 follow-ups are based on the combined sample of 484 participants (401 original at age 7 + 83 SA added at age 10)

MESCA is a population-based prospective study of childhood asthma. Participants were recruited in 1964 in Melbourne, Australia, and followed up 8 times until the most recent follow-up in their sixth decade of life. Specifically, MESCA started in 1964 when a population representative group of children born in 1957 (aged 7 years) with a history of asthma/wheeze along with controls were recruited. Participants have been subsequently followed up at the mean ages of 10, 14, 21, 28, 35, 42 and 50 years [1–8]. Participants were accompanied by their mothers for the ages 7, 10 and 14 year follow-ups and attended on their own in the subsequent studies. These follow-ups are outlined in Fig. 1 and described in detail in the section below.

#### The 1964 baseline study (mean age 7 years)

In 1964, study participants were randomly selected from 7-year-old Melbourne school children during a routine school medical examination. In this examination, the number of episodes of wheeze as well as associated respiratory tract infections over the first 7 years of life were reported by parents. From 30,000 children who completed the school medical examination, a stratified random sampling frame was used to recruit 3 wheeze/asthma groups of approximately equal size of children with a history of wheezing of varying frequency and a control group with no history of wheezing. Using different sampling ratios, 401 children were selected into four groups (Table 1): a control group (C, *n* = 106) comprised of every 20th child out of those who had never wheezed, a Mild Wheezy Bronchitis group (MWB, *n* = 75) included every second child who had less than 5 wheezing episodes associated with respiratory tract infections, a Wheezy Bronchitis group (WB, *n* = 107) included all 107 children with five or more wheezing episodes associated with respiratory tract infections and the Asthma group (A, *n* = 113) included all 113 children who had wheezed without respiratory infections on at least one occasion. The sampling method was designed to obtain representative estimates of the community prevalence of each type of wheeze. A questionnaire was completed by interviewing parents and the child’s schoolteacher. The participating children underwent a clinical examination which included pre-bronchodilator (BD) spirometry, skin prick test for common pollens, dust, and food allergens (mixed grasses, mixed inhalants, and mixed foods, rye grass, house dust, egg albumen), nasal and blood eosinophil counts, chest X-ray and histamine-binding capacity of the serum. Clinical, social, economic and behavioural data of the family were also recorded.

**Table 1:** Recruitment groups at the baseline: sample sizes and definitions

Recruitment groups	*n*	Definition
Control (C)	106	Children who had never wheezed
Mild Wheezy Bronchitis (MWB)	75	Children who had less than 5 wheezing episodes associated with respiratory tract infections
Wheezy Bronchitis (WB)	107	Children with five or more wheezing episodes associated with respiratory tract infections
Asthma (A)	113	Children who had wheezed without respiratory infections
Severe asthma (SA)*	83	Children with persistent symptoms who had an onset before three years and/or barrel-chest deformity as well as reduced lung functions with FEV_1_/FVC ≤ 50%

*this group was added at age 10 years. Five records were lost including one in C and four in MWB/WB

#### The 1967 study (mean age 10 years)

In 1967, when participants were at a mean age of 10 years, a follow-up was conducted. Of the initial 401 children, three declined participation and 19 were lost to follow-up. A total of 371 children including 94 from the control, 75 from the MWB, 100 from the WB, and 102 from the asthma groups, attended a clinical examination while parents completed questionnaires. Clinical and laboratory data similar to the baseline study were collected. A postal questionnaire was sent to 8 children and parents who had moved away from Melbourne.

During this follow-up, it was realized that very few participants had severe asthma, so further 21,000 children born in 1957 were sampled to find children with severe asthma. The severe asthma group comprised 83 children with onset of symptoms before 3 years of age, persistent symptoms at 10 years of age, barrel-chest deformity, reduction of FEV_1_/FVC ratio to 50% or less, or a combination of these factors.

#### The 1971 study (mean age 14 years)

In this follow-up, 315 of those in the wheeze/asthma groups and 82 of those in the control group were followed up at the mean age of 14 years. A similar questionnaire was completed, along with a clinical examination.

#### The 1978 study (mean age 21 years)

The next follow-up was conducted when participants were 21 years old. A total of 408 participants including 331 with asthma/wheeze and 77 controls were successfully contacted: 342 were interviewed and examined, 50 completed a questionnaire via telephone and 16 completed a postal questionnaire. Of those who did not participate, 42 were lost to follow up, 2 were deceased, out of which one death was due to asthma, and 32 were contacted but did not choose to take part.

#### The 1985 study (mean age 28 years)

A total of 371 participants were followed up at 28 years of age, including 48 controls and 323 with asthma/wheeze in childhood. A full follow-up including a questionnaire and a clinical examination (pre-BD spirometry, bronchial hyperresponsiveness (BHR), skin prick test) was undergone by 286 and a postal questionnaire was filled by 85. Blood samples were collected to measure eosinophil levels and IgE concentrations.

#### The 1992 study (mean age 35 years)

The next follow-up was conducted at the mean age of 35 years. Eleven original participants were deceased. A total of 401 participants completed this follow-up, 300 of whom physically attended and 101 who were interviewed by telephone.

#### The 1999 study (mean age 42 years)

At this follow-up, the participants were at the mean age of 42 years old, and 15 original participants were deceased, including one from asthma. A total of 403 participated in this follow-up, of these, 267 underwent a clinical examination (pre- and post- BD spirometry, BHR, skin prick test).

#### The 2005 study (mean age 50 years)

The most recent completed follow-up was conducted in 2005 when participants were 50 years old. Of the original 484 participants, 21 were deceased, two due to asthma. Five of the original records were lost, bringing the available number of participants to 458. Among these traceable participants, 34 were lost due to refusal of contact and 78 were lost to follow-up, leading to the recruitment of 346 for the study. All 346 participants completed the clinical questionnaire with a subgroup of 197 completing further objective testing (pre- and post-BD spirometry, BHR, skin prick test for 6 allergens (rye grass, dog hair, cat hair, house dust mite, egg white, and Aspergillius)).

#### The 2026 study (current follow-up)

In the current follow-up at the mean age of 69 years, we are tracing all participants from the original cohort. This follow-up includes a questionnaire and a clinical examination. In addition to respiratory health outcomes and lung function tests, we plan to do Computer Tomography (CT) scanning of the lungs and assess multimorbidity using validated questionnaires.

### What has been measured?

A clinical history, including respiratory symptoms, allergies and asthma outcomes within the past 12 months was obtained during each follow-up using standardised, study-specific questionnaires administered by trained interviewers (parent-reported in childhood, self-reported in adulthood). Information on family details including clinical, behavioural, social and economic histories were also collected. Physical examinations, lung function tests, a chest X-ray, and skin reactivity tests were conducted during the follow-ups. Spirometry was conducted according to contemporaneous standard protocols, with pre-bronchodilator testing at all follow-ups and post-bronchodilator testing in later adult follow-ups. For skin reactivity tests, scratch technique was used at 7 and 14 years and prick technique at subsequent follow-ups). BHR was assessed in those without a severe degree of airway obstruction during the adult follow-ups (exercise testing was used as the provoking agent at 21 years, methacholine at 28 years, and histamine at 35, 42 and 50 years). Blood samples were collected, and eosinophil counts of nasal and blood samples were also obtained at ages 28 to 50 years. Data collected are summarized in Tables 2 and 3.

**Table 2:** Questionnaire clinical outcome data collected in the MESCA cohort

	1964 (7 years)	1967 (10 years)	1971 (14 years)	1978 (21 years)	1985 (28 years)	1992 (35 years)	1999 (42 years)	2005 (50 years)
Asthma	√*	√*	√*	√†	√†	√†	√†	√†
Bronchitis	√*	√*	√*	√†	√†	√†	√†	√†
Hay fever	√*	√*	√*	√†	√†	√†	√†	√†
Eczema	√*	√*	√*	√†	√†	√†	√†	√†
Respiratory symptoms	√	√	√	√	√	√	√	√
Aero allergens	√*	√*	√*	√	√		√	
Food allergens	√*	√*	√*	√	√		√	
Asthma related Quality of life								√
Medication				√				√
Comorbidity							√	√

*Parent reported; † Self reported; Comorbidities=COPD, diabetes, GORD, OSA, Cardiac disease, hypertension; Medication: theophylline, steroids, bronchodilator, antihistamine

**Table 3:** Clinical assessments in the MESCA cohort

	1964 (7 years)	1967 (10 years)	1971 (14 years)	1978 (21 years)	1985 (28 years)	1992 (35 years)	1999 (42 years)	2005 (50 years)
Anthropometric measurements								
Height, Weight	√	√	√	√	√	√	√	√
Physical examination	√	√	√	√	√	√	√	√
Physical manifestations of respiratory disease							√	√
Skin reactivity tests*	√	√	√	√	√	√	√	√
Lung function measurements								
Spirometry$	√	√	√	√	√	√	√	√
Bronchial hyperreactivity (BHR)€				√	√	√	√	√
Blood investigations (Eosinophils, IgE, histamine-binding capacity of the serum)	√	√	√	√	√			
Chest X ray	√	√	√	√				

*Scratch technique at 7, 10 and 14 years and prick tests at other follow-ups; $=pre-BD at all follow-ups and post-BD at 42 and 50 years; €=exercise at 21 years, methacholine at 28 years, histamine at 35, 42 and 50 years

## Results

### Key findings and contributions

MESCA is one of a few studies in the world that have followed up children with asthma/wheeze from childhood until middle age. Similar to MESCA, the What Happens Eventually to Asthmatic children: Sociologically and Epidemiologically (WHEASE) study started in 1964 and followed up groups of children with asthma/wheeze and controls from age 10 to the sixth decade [9, 10]. In these studies, groups of asthma/wheeze were well-defined at the baseline and followed. Other longitudinal studies including the Tasmanian Longitudinal Health Study (TAHS), Dunedin and Tucson Children’s Respiratory Study (TCRS), the British National Child Development Study (NCDS), have followed the general population which included children with asthma/wheeze. The TAHS started in the same era as MESCA when all school children aged 7 years in Tasmania, including those with asthma/wheeze, were recruited in 1968 and have been regularly followed to the sixth decade [11]. The Dunedin study started in 1972 and followed children from 9 years of age to adulthood [12]. The other two relevant comparison studies are birth cohorts. The TCRS started in 1980 and followed children from birth into their fourth decade [13]. The NCDS started in 1958 and followed children born in 1958 to adulthood [14]. Together, these studies have provided insight into the natural history and outcomes of childhood asthma and wheeze with a greater emphasis on outcomes into adult life.

The main findings related to outcomes up to the sixth decade are described below. Findings from some MESCA follow-ups have been previously summarized [15].

#### Natural history of childhood asthma

**Fig. 2:**
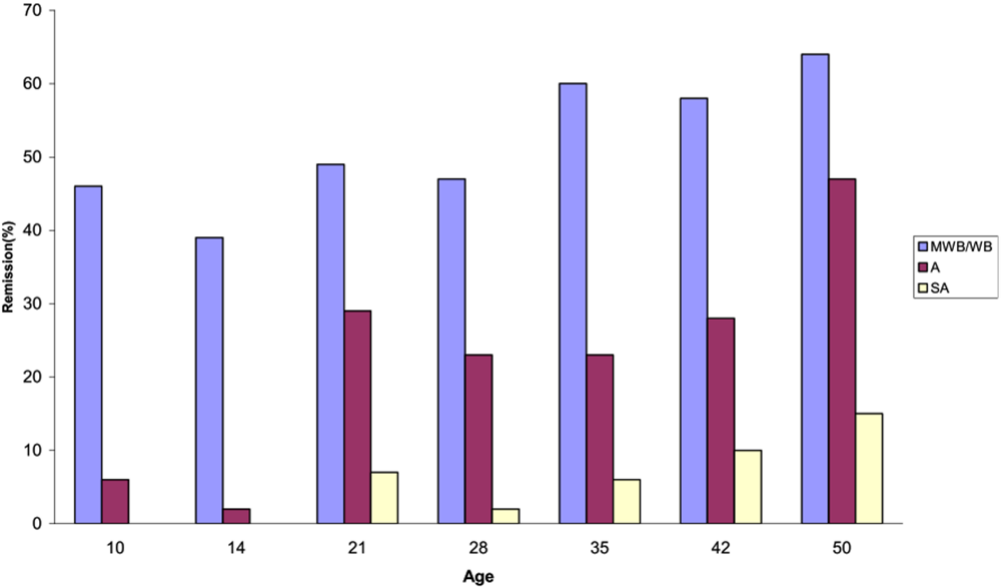
The asthma remission percentages at each review by recruitment groups. Asthma remission was defined as a subject who had no wheeze symptoms in the past 3 years and had not used bronchodilators, oral corticosteroids, or inhaled corticosteroids in the same time period. The mild wheezy bronchitis/wheezy bronchitis (MWB/WB) group has been combined. In the SA group, recruitment occurred at age 10 years, therefore, no remission described at age 10 years. Adopted with permission from ref [8]

MESCA has provided insights into the natural history of childhood asthma for the subsequent six decades. Notably, by prospectively following four asthma/wheeze groups, MESCA has been able to describe the long-term outcomes for a spectrum of severity of childhood asthma/wheeze. The proportion of remission generally increased over time in all study groups (Fig. 2) [8]. However, wheezy bronchitis was more likely to remit earlier in life while asthma/severe asthma was more likely to persist to adult life. Specifically, around 53% of the asthma and 85% of the severe asthma groups persisted to 50 years compared to 36% in the wheezy bronchitis groups [8].

These findings from MESCA are supported by those from other cohorts [9, 10] and provide evidence for the prediction of long-term outcomes for children with wheeze. Generally, the outcome is more favourable if a child only had a relatively small number of episodes of wheeze associated with a viral respiratory infection while a child with more frequent or persistent wheeze often had asthma persisting to adult life.

#### Allergies and asthma

MESCA has also provided valuable data on the associations of allergies over time for people with and without asthma/wheeze. The prevalence of current eczema significantly decreased from childhood to early adulthood (21 years) in all asthma/wheeze groups, then slightly fluctuated during middle age (Figs. S1, S2). Conversely, the prevalence of current hay fever significantly increased from childhood to adolescence (14 years) in all groups except the severe asthma group [16], then became relatively stable. These findings suggest that the childhood through adolescence period is a critical phase for transition in allergies.

Collecting data on both asthma/wheeze and allergies over time has enabled MESCA to tease out the role allergies play in the course of childhood asthma/wheeze. In children with asthma/wheeze, having an atopic condition (i.e. hay fever, eczema or positive skin test reactivity) in childhood was predictive of more severe asthma in adult life. Conversely, the severity of childhood asthma/wheeze was associated with increased risk of hay fever and eczema in later life [6].

#### Lung function change over time

**Fig. 3:**
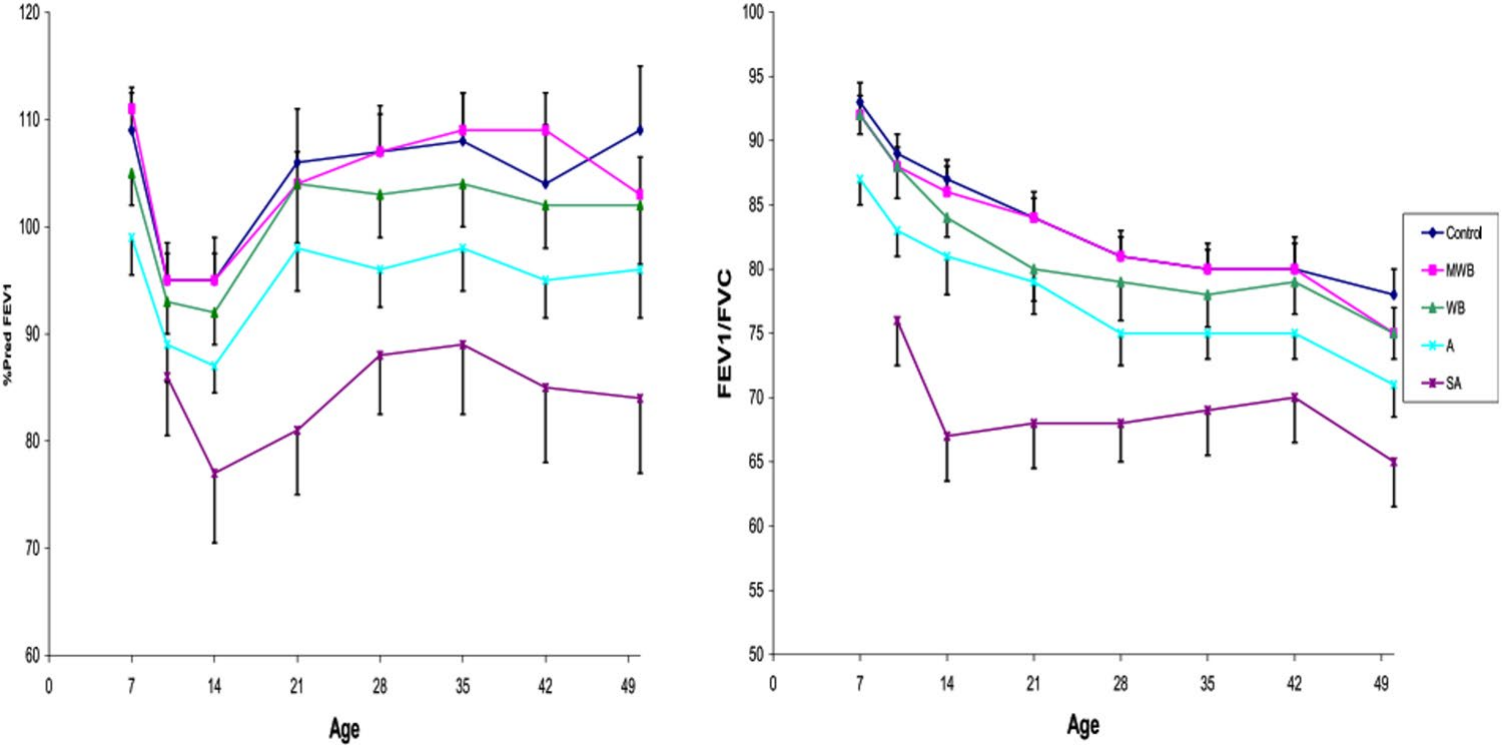
FEV_1_ (% predicted) and FEV_1_-FVC (%) at each review by classification at recruitment. Mean ± 95th CI. Adopted with permission from ref [8]

Having uniquely extensive longitudinal lung function data spanning six decades allows the MESCA study to delineate lung function trajectories over time associated with childhood asthma. The MESCA cohort demonstrated that lung function tracked from childhood into adulthood across all study groups (Fig. 3, S3). In particular, participants with mild wheezy bronchitis or wheezy bronchitis had similar FEV_1_ and FEV_1_/FVC levels over time compared to the controls while those with asthma or severe asthma had ongoing airways obstruction (reduced FEV_1_ and FEV_1_/FVC). Indeed, participants in the severe asthma group had the lowest level of lung function, which was already evident by age 14 years. Although asthma and severe asthma groups had reduced lung function levels over time compared to the control or wheezy bronchitis groups, their rates of lung function decline were relatively similar.

The tracking of lung function over time observed in MESCA is consistent with findings from other cohorts. The WHEASE study similarly reported persistently lower lung function levels, but no evidence of accelerated lung function decline, among their wheezing groups [10].

As previously described [15], the lung function trajectories of the asthma/wheeze groups observed in MESCA raise interesting questions. The early loss of lung function in the severe asthma group suggests that adequate/optimal early treatment with anti-inflammatory medication for severe childhood asthma might have prevented early lung function deterioration and subsequent long-term lung function damage. Oral corticosteroids were available from the 1950 s, but the use of inhaled corticosteroids was not widespread until the 1970s. On the other hand, children with infrequent wheeze still had the more optimistic lung function outcome into adulthood, which highlights that symptom control should be the primary treatment goal for these children rather than preventing accelerated loss of lung function.

#### The association between childhood asthma and COPD in middle age

One of the key findings of MESCA was that it is the first prospective study to provide robust evidence for the link between childhood asthma and the development of COPD. Specifically, this study revealed that children with asthma, and particularly severe asthma at baseline had a 10- to 32-fold increase in risk of COPD by 50 years of age [17].

This novel finding from MECSA is confirmed by other studies. The WHEASE cohort reported that individuals with childhood asthma had a six- fold increased risk of COPD by age 61 years [10]. Similarly, the TAHS cohort demonstrated associations between distinct childhood asthma profiles and an increased risk of COPD by age 53 years [18]. Taken together, these findings underscore childhood asthma as a strong risk factor for later-life COPD and highlight opportunities for COPD prevention, including optimal long-term control of childhood asthma across the life course.

COPD can stem from different lung function trajectories comprising accelerated lung function decline and/or reduced lung function before adulthood [19, 20]. Findings of the MESCA study highlight that COPD related to childhood asthma may be more predominantly characterised by impaired lung function that tracks along a similar percentile over time rather than featuring an accelerated lung function decline during adulthood.

## Discussion

### Limitations

The natural history and long-term outcomes of childhood asthma/wheeze described in MESCA and studies conducted in the same era are relevant but may slightly differ from the current context. MESCA’s participants were born in 1957 and treatment practice has changed over time. In 1960 s, treatment of asthma with corticosteroid therapies was often inadequate compared to current practice. Anti-inflammation medication was not routinely used for asthma/wheeze and antibiotics used to be preferentially prescribed for wheezy bronchitis. Thus, findings of MESCA and studies in the same era could be complemented by long-term follow-ups of more recent cohorts as data become available.

In summary, while extensive longitudinal studies of childhood health and illness present challenges, they offer valuable insights across the life course. MESCA exemplifies this, with participants now entering their seventh decade of life. Their rich, lifetime data provides a unique opportunity to investigate a wide range of outcomes, including multimorbidity and healthy aging.

### Can I get hold of the data?

All data are stored at a secured research server located at the University of Melbourne. Researchers interested in collaboration should contact Allergy and Lung Health Unit, School of Population and Global Health, the University of Melbourne.

## Supplementary Information

Below is the link to the electronic supplementary material.

Supplementary Material 1
