# Supplementary material for: Melbourne Epidemiological Study of Childhood Asthma (MESCA)
Source: Eur J Epidemiol. 2026 Mar 18;41(4):519–25. doi: 10.1007/s10654-026-01377-3 (PMC13331842; doi:10.1007/s10654-026-01377-3)
Supplement: Supplementary file 1 — Supplementary Material 1 [file 10654_2026_1377_MOESM1_ESM.docx]

Online Supplement

**Cohort profile: Melbourne Epidemiological Study of Childhood Asthma (MESCA)**

^#^Bui DS^1^, ^#^Tai A^2,3^, Liu J^1^, Perret J^1^, Lodge C^1^, Idrose NS^1^, Lowe A^1^, *Robertson C^4^, *Dharmage SC^1^


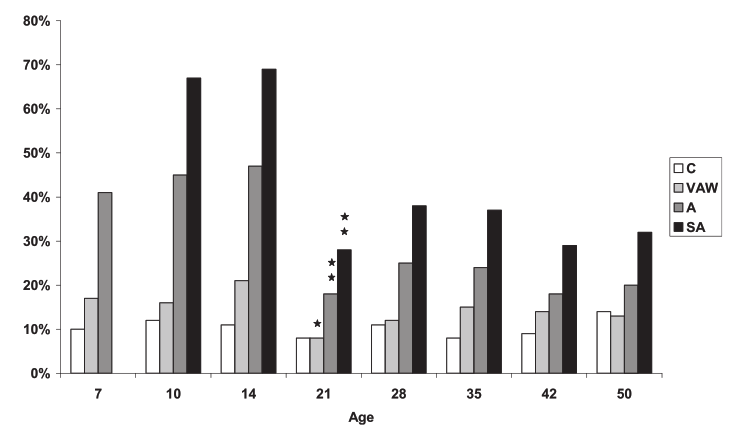


**Fig S1**: Prevalence of eczema in the past 12 months at each period by recruitment groups (*P < .05, ** P < .001). A, asthma; C, control (no asthma); SA, severe asthma; VAW, viral-associated wheeze. Adopted with permission from ref 16


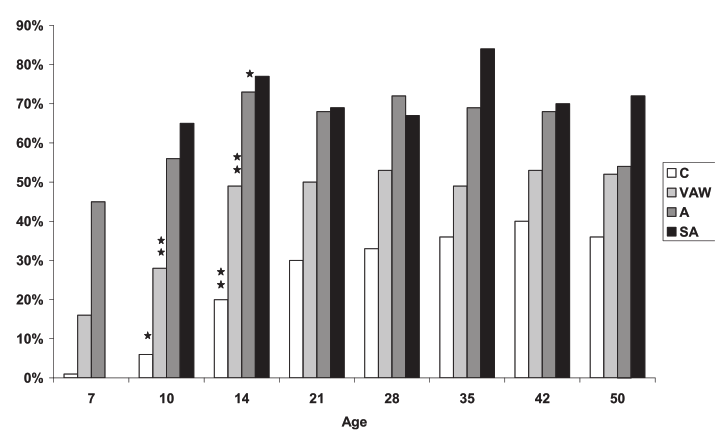


**Fig S2**: Prevalence of allergic rhinitis in the past 12 months at each period by recruitment groups (*P < .05, ** P < .01). A, asthma; C, control (no asthma); SA, severe asthma; VAW, viral-associated wheeze. Adopted with permission from ref 16


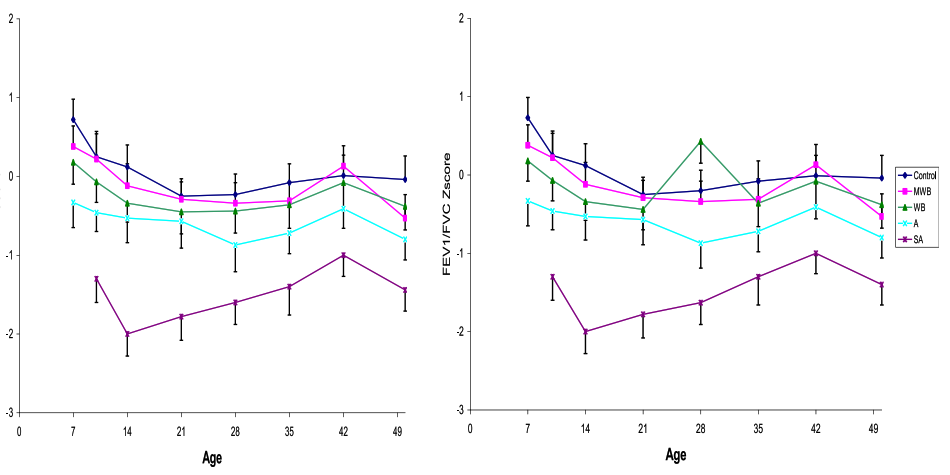


**Fig S3**. FEV_1_ and FEV_1_-FVC at each review by classiﬁcation at recruitment presented as z scores. Mean± 95th CI. Adopted with permission from ref 8
